# Incidence of Pelvic Ring Fractures in the U.S. Military Population

**DOI:** 10.7759/cureus.6899

**Published:** 2020-02-06

**Authors:** John J Pisquiy, Jordan T Carter, Andrew Chan, Nicholas Kusnezov, Adam Adler

**Affiliations:** 1 Orthopaedic Surgery, Texas Tech University Health Science Center, El Paso, USA; 2 Orthopaedics, Texas Tech University Health Sciences Center, El Paso, USA; 3 Orthopaedic Surgery, William Beaumont Army Medical Center, El Paso, USA

**Keywords:** epidemiology, military, pelvic fractures, incidence

## Abstract

Introduction

Pelvic ring fractures occur frequently among the elderly population, but some studies demonstrate a bimodal distribution where the incidence is elevated among younger age groups as well. The mechanisms of injury also vary based on age groups. Previous studies are specific to trauma registries and centers, but epidemiological data within the U.S. military are sparse. In the U.S. military population, pelvic ring fractures can be related to high-energy trauma including motor vehicle accidents and combat warfare. The purpose of this study was to determine the incidence of pelvic ring fractures among active duty U.S. military service members between 2006 and 2015, while also describing the demographics associated with the findings.

Materials and Methods

All data were collected from the U.S. Defense Medical Epidemiology Database (DMED). To calculate the incidence rates, only first-time occurrences for pelvic ring fractures among military members were used. Each point was identified using International Classification of Diseases, Ninth Revision (ICD-9), clinical modification code 808 for “fractures of the pelvis.” A multivariate Poisson regression analysis was used to estimate the incidence rate per 1,000 person-years and 95% confidence intervals while controlling for sex, race, age, rank, and service. Rate ratios were calculated using different referent factors based on differences in sex, race, age, rank, and service branch. This study was IRB exempt as all the data used were de-identified patient data from the DMED system.

Results

Over the 10-year study period, a total of 4,802 incident cases of pelvic ring fractures, and a total of 13,748,429 person-years were documented. The overall incidence rate of pelvic ring fractures was 0.35 per 1,000 person-years. The incidence of pelvic ring fractures was highest among the youngest age group (<20 years) and among the lower-ranking service members. Additionally, other demographic groups such as the White race, female sex, and Army service members showed the highest incidence rates.

Conclusion

Our study determined baseline epidemiological data on incidence rates of pelvic ring fractures in the U.S. military. Patient demographics may be contributing factors, and the present analysis was able to elucidate associated underlying demographics. We demonstrated that the incidence was highest among the younger age groups, and that incidence rates may be specific to age cohorts. This study also found that lower-ranking service members had the highest incidence in all service branches, suggesting a form of occupational risk. Furthermore, our findings suggest that females, White race groups, and Army enlisted service members show a significantly higher incidence rate and may be at a greater risk. Our findings are important as they broaden the understanding of the patterns of pelvic ring fractures in the U.S. military population and occupational risks associated with this population.

## Introduction

Pelvic ring fractures can result from different mechanisms of injury, such as low-impact falls among the elderly population, and high-energy, traumatic events, such as motor vehicle accidents (MVAs) [1,2]. Pelvic ring fractures are high-risk injuries that require careful evaluation due to significant patient morbidity and mortality associated with damage to major blood vessels, nerves, and organs [2]. Among the military population, the increased use of improvised explosive devices (IEDs) against U.S. soldiers in Iraq and Afghanistan is an additional risk factor that has lead to an increased incidence of complex traumatic pelvis injuries [3].

Previously reported incidence rates have varied significantly (0.13-9.35 per 1,000 person-years) depending on the study populations [4-7]. Some studies demonstrate a bimodal distribution of the elevated incidence among the elderly (>65 years) and younger age groups (20-40 years) based on different mechanisms of injury. Among a young active population, epidemiological evidence for pelvic ring fractures is not well elucidated, especially when referencing the U.S. military.

The goal of this study was to report the incidence rates of pelvic ring fractures and epidemiological variables found in all U.S. Military service members. Epidemiological surveillance and reports are important as they can broaden the understanding of injury patterns and allow for proper resource allocation for their prevention [8]. The data from these studies can also lead to enhanced treatment, preventive strategies, and the provision of trauma services [9]. We hypothesized that the younger US military population would have a higher incidence of pelvic ring fractures than the older military population overall.

## Materials and methods

Current and historical data regarding medical events (e.g. hospitalizations, ambulatory visits, reportable diseases) and demographic information (e.g. sex, race, age, rank, service branches) of the US Armed Forces are housed within the Defense Medical Surveillance System (DMSS). The DMSS integrates standardized data from multiple Department of Defense services worldwide. The Defense Medical and Epidemiology Database (DMED) application provides access to these archives by using user-definable queries for International Classification of Diseases, Ninth Revision (ICD-9) coding information for every patient encounter in a military treatment facility [10].

Only the ambulatory and in-patient hospitalization data were used in this study to determine the incidence of pelvic ring fractures between the years 2006 and 2015. In the DMED system, the pelvic ring fracture data were queried using the ICD-9 Clinical Modification Code 808 for “fracture of pelvis.” 

Collected data were limited to first occurrences and were categorized according to patient demographics. Both the ambulatory and hospitalization data were totaled by year and by subset patient demographics.

A multivariable Poisson regression was used to estimate the rate of pelvic ring fractures per 1,000 person-years by sex, race, age, rank, and service. We also computed rate ratios for sex, using males as the referent, and controlling for differences in race, age, rank, and service between males and females (adjusted rates). Rate ratios were also calculated for race, using Whites as the referent category; age, using <20 years as the referent category; rank, using Jr. officers as the referent category; and service, using the Air Force as the referent category, all of which were adjusted for other covariates. All of the data used are de-identified patient data from the DMED system, and our study underwent an IRB exemption process.

## Results

Between 2006 and 2015, a total of 4,802 pelvic ring fractures occurred in our population at risk of 13,748,491 person-years. The overall incidence rate of pelvic ring fractures was 0.35 per 1,000 person-years (95% confidence interval [95% CI] 0.34-0.36).

Among the different age groups, the highest crude incidence rate was among the <20 year age group at a rate of 0.59 per 1,000 person-years. When adjusting for gender, race, rank, and service, the <20 year age group continued to have the highest risk of pelvic ring fractures. The 30-34 year age group had the lowest adjusted incidence rate ratio (IRR) of 0.63 (95% CI 0.55-0.72) when using the <20 year age group as the referent group. The 20-24 year age group had the highest adjusted rate ratio of 0.82 (95% CI 0.75-0.91), using the <20 year age group as the referent group (Table 1). All findings were statistically significant.

**Table 1:** Unadjusted Incidence Rates and Adjusted Incidence Rate Ratios (IRRs) of Pelvic Ring Fractures Among Military Service Members, 2006-2015, by Age Group * Incidence rate is per 1,000 person-years ‡ Adjusted for gender, race, rank, and service. <20 year age group was the referent group.

Age group, years	Cases	Person-years		Unadjusted IR*	Adjusted IRR (95% CI)
<20	519		883119	0.59	1
20-24	1927		4455654	0.43	0.82 (0.75,0.91) ^‡^
25-29	1048		3264541	0.32	0.74 (0.65,0.83) ^‡^
30-34	511		2084300	0.25	0.63 (0.55,0.72) ^‡^
35-39	418		1603068	0.26	0.72 (0.62,0.84) ^‡^
≥40	379		1457809	0.26	0.77 (0.65,0.90) ^‡^

In evaluating the differences among sex, females and males had unadjusted incidence rates of 0.59 and 0.31 per 1,000 person-years, respectively. When adjusting for age, race, rank, and service, females had a higher adjusted rate ratio of 2.22 (95% CI 2.07-2.37), when using males as the referent group (male adjusted rate ratio of 1). These findings were statistically significant.

Analyzing the data based on race, the White, Black, and other race groups had unadjusted incidence rates for pelvic ring fractures per 1,000 person-years of 0.37, 0.31, and 0.29, respectively. When using the White race group as the referent group (White adjusted rate ratio of 1), the other and the Black race group showed adjusted rate ratios of 0.79 (95% CI 0.72-0.86) and 0.71 (95% CI 0.65-0.77), respectively, when controlling for age, gender, rank, and service. When stratifying the race data by age groups, the White, <20 year age group had the highest crude incidence rate of 0.62 (Table 2). When using the White race as the referent group, the other, 20-24 year age group had the highest risk with a risk ratio of 92% (95% CI, 0.80-1.06) (Table 2) for pelvic ring fractures. All findings were statistically significant.

**Table 2:** Unadjusted Incidence Rates and Adjusted Incidence Rate Ratios (IRRs) of Pelvic Ring Fractures Among US Military Service Members, 2006-2015, by Age and Race N/A = not applicable since this category was used as the referent category for the calculations. * Incidence rate is per 1,000 person-years. ‡White was the referent category. Adjusted for age, gender, rank, and service.

Category	Cases	Person-years	Unadjusted IR*	Adjusted IRR (95% CI) ‡
< 20				
White	404	649902	0.62	N/A
Other	49	103362	0.47	0.82 (0.61,1.11)^‡^
Black	66	129855	0.51	0.75 (0.58, 0.98)^‡^
20-24				
White	1459	3212566	0.45	N/A
Other	226	573729	0.39	0.92 (0.80, 1.06)^‡^
Black	242	669359	0.36	0.70 (0.61, 0.81)^‡^
25-29				
White	768	2253302	0.34	N/A
Other	100	479298	0.21	0.61 (0.49, 0.75)^‡^
Black	180	531941	0.34	0.81 (0.69, 0.95)^‡^
30-34				
White	351	1369855	0.26	N/A
Other	76	328344	0.23	0.83 (0.65, 1.07)^‡^
Black	84	386101	0.22	0.66 (0.52, 0.84)^‡^
35-39				
White	281	1042267	0.27	N/A
Other	52	229531	0.23	0.72 (0.54, 0.97)^‡^
Black	85	331270	0.26	0.71 (0.55, 0.90)^‡^
≥40				
White	274	982865	0.28	N/A
Other	45	189483	0.24	0.70 (0.51, 0.96)^‡^
Black	60	285461	0.21	0.51 (0.38, 0.68)^‡^
Overall				
White	3537	9510757	0.37	N/A
Other	548	1903747	0.29	0.79 (0.72, 0.86)^‡^
Black	717	2333987	0.31	0.71 (0.65, 0.77)^‡^

Military ranks were also assessed in analyzing the incidence of pelvic ring fractures. By rank, the Jr. enlisted group (E1-E4) had the highest crude incidence rate of 0.46 per 1,000 person-years. Sr. enlisted (E5-E9), Jr. officer (O1-O3), and Sr. officer (O4-O9) groups had unadjusted incidence rates of 0.28, 0.21, and 0.20 per 1,000 person-years, respectively. When using the Jr. officer group as the referent group (Jr. enlisted adjusted rate ratio of 1), the Jr. enlisted, Sr. enlisted, and Sr. officer groups had adjusted rate ratios of 1.91 (95% CI 1.68-2.17), 1.51 (95% CI 1.33-1.71), and 0.98 (95% CI 0.80-1.19), respectively, when adjusting for age, gender, race, and service. All findings were statistically significant. When stratifying the data by age groups, there were no adjusted rate ratios that could be calculated as there were multiple rank groups within each age group that did not have any incident cases of pelvic ring fractures.

Among each service branch, the Army, Navy, Marines, and Air Force had crude incidence rates of 0.47, 0.23, 0.46, and 0.20 per 1,000 person-years, respectively. When using the Air Force as the referent group (Air Force adjusted rate ratio of 1), and controlling for age, sex, race, and rank, the Army, Navy, and Marines had adjusted IRRs of 2.45 (95% CI, 2.25-2.68), 1.18 (95% CI 1.06-1.31), 2.22 (95% CI 2.00-2.46), respectively. Additionally, when stratifying the data by age groups, service members who were <20 years and in the Army had the highest unadjusted incidence rates of 0.83 per 1,000 person-years. The Army service branch had the highest adjusted IRRs followed by the Marine service branch in all age groups (Table 3). All findings were statistically significant and adjusted for age, gender, race, and rank.

**Table 3:** Unadjusted Incidence Rates and Adjusted Incidence Rate Ratios (IRRs) of Pelvic Ring Fractures Among US Military Service Members, 2006-2015, by Age and Service N/A = not applicable since this category was used as the referent category for the calculations. * Incidence rate is per 1,000 person years. ‡Air Force was the referent category. Adjusted for age, gender, rank, and service.

Category	Cases	Person-years	Unadjusted IR*	Adjusted IRR (95% CI)
< 20				
Army	254	307747	0.83	2.82 (2.05, 3.89)^ ‡^
Navy	68	184393	0.37	1.22 (0.83, 1.78)^ ‡^
Marines	153	248901	0.61	2.23 (1.59, 3.12)^ ‡^
Air Force	44	142078	0.31	N/A
20-24				
Army	908	1604146	0.57	2.24 (1.95, 2.58)^ ‡^
Navy	296	1031116	0.29	1.12 (0.94, 1.32)^ ‡^
Marines	473	896737	0.53	2.15 (1.84, 2.51)^ ‡^
Air Force	250	923655	0.27	N/A
25-29				
Army	571	1274638	0.45	2.62 (2.18, 3.14)^ ‡^
Navy	176	787114	0.22	1.32 (1.06, 1.64)^ ‡^
Marines	154	377049	0.41	2.57 (2.05, 3.23)^ ‡^
Air Force	147	825740	0.18	N/A
30-34				
Army	292	838089	0.35	2.69 (2.08, 3.47)^ ‡^
Navy	88	504718	0.17	1.34 (0.99, 1.83)^ ‡^
Marines	56	187256	0.3	2.53 (1.79, 3.58)^ ‡^
Air Force	75	554237	0.14	N/A
35-39				
Army	249	643820	0.39	2.20 (1.71, 2.82)^ ‡^
Navy	54	398320	0.14	0.76 (0.54, 1.08)^ ‡^
Marines	33	127229	0.26	1.61 (1.07, 2.41)^ ‡^
Air Force	82	433699	0.19	N/A
≥40				
Army	234	618530	0.38	2.70 (2.03, 3.60)^ ‡^
Navy	68	351855	0.19	1.41 (0.99, 1.99)^ ‡^
Marines	18	88285	0.2	1.61 (0.95, 2.73)^ ‡^
Air Force	59	399139	0.15	N/A
Overall				
Army	2508	5286970	0.38	2.45 (2.25, 2.68)^ ‡^
Navy	750	3257516	0.19	1.18 (1.06, 1.31)^ ‡^
Marines	887	1925457	0.2	2.22 (2.00, 2.46)^ ‡^
Air Force	657	3278548	0.15	N/A

## Discussion

The etiology of pelvic ring fractures can vary from low-energy injuries associated with osteoporosis to high-energy injuries that cause life-threatening, unstable fractures [2]. Different mechanisms of injury have led to peaks of incidence among two different populations (e.g. elderly and trauma-related populations). Previous studies have focused on these two groups separately in their analyses.

The low-energy fractures are associated with the elderly population, as age-related problems such as falling and osteoporosis are the main causes of injury to this population [11-13]. Reported data of pelvic ring fractures among older age groups have increased dramatically over the past decades with reported incidence rates ranging from 0.13 to 2.52 per 1,000 person-years [1,4,5,11]. Trauma-related injuries vary based on civilian and military causes. The most common mechanism of civilian trauma is MVAs [8]. Military causes of pelvic ring fractures include IEDs, non-IED explosions, MVAs, and gunshot wounds. Among civilian trauma, reported incidence rates are largely unknown. In a 12-month study in Australia, the reported incidence rate was 0.23 per 1,000 person-years [6]. In another U.S. study, researchers reported incidence rates of open versus closed pelvic fractures to be between 7.6% and 92.4% when analyzing 24,059 patients with unstable pelvic ring fractures, respectively, using ICD-9 Clinical Modification Codes [14]. In respect to military trauma, a U.K. Military study reported an overall incidence rate of 7.4% among dismounted soldiers admitted to a field hospital due to blast injuries sustained from IED explosions [15]. Another study using U.K. Military Joint Theatre Trauma Registry reported a total incidence of pelvic ring fractures (both open and closed) to be 0.83 per 1,000 person-years [16]. Epidemiology over the U.S. military population regarding pelvic ring fractures is limited. Reported incidence rates among all age groups are sparse, and available literature focuses on patient outcomes, hospital management, and the epidemiology of the elderly population [1,3,7,8,11,12,17-19].

Patient demographics may be contributing factors, and our analysis sought to elucidate these underlying demographics and determine the incidence of pelvic ring fractures among a young, active military population. Our pelvic ring fracture incidence of 0.35 per 1,000 person-years (95% CI, 0.34-0.36) lies in between previously reported incidence rates. However, the U.S. military population of young, active, predominantly male individuals contrasts with the typical adult population that may have lesser physical and functional demands [20-22].

Among the age groups, the highest rates of pelvic ring fractures were found in the <20 and 20-24 year age groups with crude incidence rates of 0.59 and 0.43 per 1,000 person-years. Adjusted rate ratios for the <20 and 20-24 year age groups correlated with ratios of 1.0 and 0.82 per 1,000 person-years, respectively (Table 1). These results correspond with our hypothesis and could be attributed to younger service members being more likely to be combat service members (i.e. dismounted warfighters) that are exposed to ground-emplaced IEDs [15]. In a study about dismounted blast injuries, the median age of service members was 23 years [15]. In addition, the second-highest incidence rate in the 20-24 year age group corresponds to our hypothesis. While the ≥40 year age group is very broad, other studies over the elderly population have shown that incidence generally increases with age [1,7,11-13].

Females had a 2.22 times higher risk of pelvic ring fractures than males when adjusting for age, race, rank, and service. This higher incidence in females corresponds with data found in studies over the elderly population [1,7,11-13]. In addition, past data have shown that pelvic ring fractures are more common among female recruits compared with their male counterparts [23-25]. The female gender is generally understood to be a risk factor for bone stress injuries, especially via non-traumatic mechanisms [26,27].

In evaluating the incidence by race, the White race group had the highest crude incidence rate among all other race groups. When adjusting for age, sex, rank, and service, the "other" and Black race groups had a decreased risk of 21% and 29%, respectively, when using Whites as the referent group. This finding is significant as the "other" race group only makes up approximately 11% of the total number of pelvic ring fracture cases while the White and Black groups make up approximately 74% and 15%, respectively (Table 3). To our knowledge, there are no other studies that look specifically at race as a potential risk factor for pelvic ring fractures. We acknowledge that the analysis of the "other" race group is limited considering that it combines all other racial groups. When stratified by age, all but the 25-29 year age group showed that the "other" race group had the highest incidence rate of pelvic ring fractures (Table 2).

Our study assumed a difference in occupational risk based on military rank. We assumed that lower-ranked, enlisted service members were generally exposed to more MVAs and combat explosions. Higher-rank service members were least likely to be exposed to traumatic events because they were more likely to have officer administrative duties. Additionally, service members on foot patrol are more likely to have a specific pattern of injuries due to IEDs compared to other service members [3,16]. In this study, the highest crude incidence rate of 0.46 per 1,000 person-years was found in the Jr. enlisted group. The highest adjusted incidence ratio of 1.91 (95% CI 1.68-2.17) was found in the Jr. enlisted group, when using the Jr. officer group as the referent group. Subsequent groups decreased in incidence rates with increased rank. This correlation may be due to the increased combat exposure among the younger enlisted service members. This not a definitive finding, as further studies are recommended to help further explain the higher incidence in the Jr. enlisted group and reveal other potential occupational risk factors.

Among the service branches, the highest incidence rates were found in the Army, subsequently followed by the Marines. The highest adjusted rate ratios were also found in the Army, followed by the Marines. Service branches also serve as a crude measurement of occupational risk. Within each military branch, service members are assigned to different military occupational specialties (MOSs), and each MOS has different physical and medical standards. However, the details of the different physical requirements of each MOS are not clear [15]. In our stratified analysis by age, the Army had the highest unadjusted incidence rates and adjusted ratios, followed by the Marines, in all age groups. The highest rates in the Army were found among the <20 year age group and the 20-24 year age groups, subsequently (Table 3).

Our study has several limitations. The utilization of the DMED application poses a significant limitation as all the data included were obtained by using ICD-9 diagnostic codes. While these codes are useful in data tracking, they are subject to error and inaccuracy due to variance in electronic and written records, misspecification, and miscoding as all physicians and mid-level providers can make a diagnosis [28]. Another limitation was the use of ICD-9 Code 808 for pelvic ring fractures. This code is specific to “fractures of the pelvis,” but it does not delineate the type of pelvic ring fracture (e.g. acetabulum closed or open, pubis closed or open, ischium closed, and ilium closed) in each occurrence. During the coding process, any type of pelvic ring fracture may be included. Another limitation could be the discrepancies in diagnostic criteria used by the physicians when logging the entry. A physician may over-report or under-report the incidence of pelvic ring fractures based on the diagnostic code used. In our study, we were unable to retrospectively investigate specific diagnostic criteria (e.g. mechanism of injury, surgical dictations) used in each patient encounter. External validity may also pose a limitation to this study as the military population (e.g. younger, more active, and predominately male) is distinctly different from a general civilian population. Finally, our study could not control or evaluate occupational risk although we assumed different risks among different ages, ranks, and military branches.

## Conclusions

This study provides baseline epidemiological data of pelvic ring fractures in the U.S. military population. Through our investigation, we found that pelvic ring fractures may have a higher incidence in specific age cohorts. Furthermore, our findings suggest that females, White race groups, and junior enlisted service members show a significantly higher incidence rate and may be at a greater risk. The findings presented in our military analysis provide epidemiological data that can enhance the understanding of injury patterns within this specific cohort.
